# Evaluation of a National Comprehensive Cancer Network Guidelines–Based Decision Support Tool in Patients With Non–Small Cell Lung Cancer: A Nonrandomized Clinical Trial

**DOI:** 10.1001/jamanetworkopen.2020.9750

**Published:** 2020-09-30

**Authors:** Susan Y. Wu, Ann A. Lazar, Matthew A. Gubens, Collin M. Blakely, Alexander R. Gottschalk, David M. Jablons, Thierry M. Jahan, Victoria E. H. Wang, Taylor L. Dunbar, Melisa L. Wong, Jason W. Chan, William Guthrie, Jeff Belkora, Sue S. Yom

**Affiliations:** 1 Department of Radiation Oncology, University of California, San Francisco; 2 Department of Epidemiology and Biostatistics, University of California, San Francisco; 3 Division of Hematology/Oncology, Department of Medicine, University of California, San Francisco; 4 Department of Surgery, University of California, San Francisco; 5 PatientsWithPower Inc, Santa Clara, California; 6 Philip R. Lee Institute for Health Policy Studies, University of California, San Francisco

## Abstract

**Importance:**

The association of guideline-based decision support with the quality of care in patients with non–small cell lung cancer (NSCLC) is not known.

**Objective:**

To evaluate the association of exposure to the National Comprehensive Cancer Center (NCCN) guidelines with guideline-concordant care and patients’ decisional conflict.

**Design, Setting, and Participants:**

A nonrandomized clinical trial, conducted at a tertiary care academic institution, enrolled patients from February 23, 2015, to September 28, 2017. Data analysis was conducted from July 19, 2019, to April 22, 2020. A cohort of 76 patients with NSCLC seen at diagnosis or disease progression and a retrospective cohort of 157 patients treated before the trial were included. Adherence to 6 NCCN recommendations were evaluated: (1) smoking cessation counseling, (2) adjuvant chemotherapy for patients with stage IB to IIB NSCLC after surgery, (3) pathologic mediastinal staging in patients with stage III NSCLC before surgery, (4) pathologic mediastinal staging in patients with stage III NSCLC before nonsurgical treatment, (5) definitive chemoradiotherapy for patients with stage III NSCLC not having surgery, and (6) molecular testing for epidermal growth factor receptor and anaplastic lymphoma kinase alterations for patients with stage IV NSCLC. Subgroup analysis was conducted to compare the rates of guideline concordance between the prospective and retrospective cohorts. Secondary end points included decisional conflict and satisfaction.

**Interventions:**

An online tool customizing the NCCN guidelines to patients’ clinical and pathologic features was used during consultation, facilitated by a trained coordinator.

**Main Outcomes and Measures:**

Concordance of practice with 6 NCCN treatment recommendations on NSCLC and patients’ decisional conflict.

**Results:**

Of the 76 patients with NSCLC, 44 were men (57.9%), median age at diagnosis was 68 years (interquartile range [IQR], 41-87 years), and 59 patients (77.6%) had adenocarcinoma. In the retrospective cohort, 91 of 157 patients (58.0%) were men, median age at diagnosis was 66 years (IQR, 61-65 years), and 105 patients (66.9%) had adenocarcinoma. After the intervention, patients received more smoking cessation counseling (4 of 5 [80.0%] vs 1 of 24 [4.2%], *P* < .001) and less adjuvant chemotherapy (0 of 7 vs 7 of 11 [63.6%]; *P* = .012). There was no significant change in mutation testing of non–squamous cell stage IV disease (20 of 20 [100%] vs 48 of 57 [84.2%]; *P* = .10). There was no significant change in pathologic mediastinal staging or initial chemoradiotherapy for patients with stage III disease. After consultation with the tool, decisional conflict scores improved by a median of 20 points (IQR, 3-34; *P* < .001).

**Conclusions and Relevance:**

The findings of this study suggest that exposure to the NCCN guidelines is associated with increased guideline-concordant care for 2 of 6 preselected recommendations and improvement in decisional conflict.

**Trial Registration:**

ClinicalTrials.gov Identifier: NCT03982459

## Key Points

**Question:**

Is exposure to the National Comprehensive Cancer Center guidelines associated with decreased decisional conflict and increased rates of guideline-concordant care in patients with non–small cell lung cancer?

**Findings:**

In this nonrandomized clinical trial, exposure to the National Comprehensive Cancer Center guidelines in 76 patients with non–small cell lung cancer was associated with increased smoking cessation counseling and decreased use of adjuvant chemotherapy after resection of early-stage disease. Use of the tool during consultation was also associated with decreased decisional conflict and greater satisfaction with their decision by the patients.

**Meaning:**

The findings of this study suggest that use of cancer treatment guidelines is not in conflict with shared decision-making; increasing patients’ access to guidelines appears to improve the quality of oncologic care.

## Teaser

This nonrandomized clinical trial examines the inclusion of National Comprehensive Cancer Network (NCCN) guidelines in a tool used by patients with non–small cell lung cancer to facilitate decision-making.

## Introduction

Patients with non–small cell lung cancer (NSCLC) navigate a complicated sequence of diagnostic tests and choices of treatment and are often faced with a complex decision-making framework that evolves substantially through the course of their workup. National treatment guidelines are not easily accessible for these patients, whose treatment preferences may change based on upstaging, individual factors, or personal circumstances.^1^

This burden of decision-making is stressful. Decisional conflict is associated with delays in decision-making, vacillation between treatment options, and regret.^2,3,4^ Decision support tools can improve patient knowledge regarding options, prognosis, and selection of treatment that best balances their goals of care,^5,6^ but most tools have been developed for patients with breast^6,7^ and prostate cancer or advanced/metastatic cancer.^8,9,10^ Furthermore, to our knowledge, there have been no studies demonstrating changes in practice patterns based on enhanced decision support in patients with NSCLC.

The aim of this clinical trial was to evaluate an interactive web-based tool that allows patients to explore individually tailored decision trees derived from the National Comprehensive Cancer Center (NCCN) guidelines. The primary end point was the association with predetermined benchmarks of guideline-concordant quality care. Changes in decisional conflict, satisfaction with decisions, and other metrics of patient experience were also assessed.

## Methods

### Patient Selection

Physicians in the multidisciplinary lung cancer practice identified eligible patients scheduled for consultation. Trained research coordinators then screened these patients, identifying 84 eligible patients, of whom 76 were enrolled (90% enrollment). Patients were older than 18 years; able to use an English-language, web-based interface; and had NSCLC that was either newly diagnosed and treatment naive or newly recurrent or progressive, requiring a change in therapy. Eligible histologic types included adenocarcinoma, squamous cell, large cell, and adenosquamous carcinoma; patients with radiographically diagnosed NSCLC who were unable to undergo biopsy were also eligible. Clinical data, including staging, treatments received, and demographic information, were abstracted from the medical record. Enrolled patients retained access to the web-based tool until trial closure and consented to have their use monitored for 1 year.

This trial was approved by the institutional review board of the University of California, San Francisco, and participants in the prospective cohort provided written informed consent; aside from access to the web-based tool, there were no incentives to participate. Consent for retrospective data collection was waived by the institutional review board. The retrospective data set comprised patients who had consultations with the same group of physicians within the 8 months preceding the trial and met the same eligibility criteria. The NCCN guidelines v1.2014 were used to program the web-based tool and evaluate guideline concordance in the retrospective study cohort.^11^ The trial protocol is available in Supplement 1. This study followed the Transparent Reporting of Evaluations With Nonrandomized Designs (TREND) reporting guideline for nonrandomized clinical trials.

### Guideline Tool and Assessments

**Figure 1.**
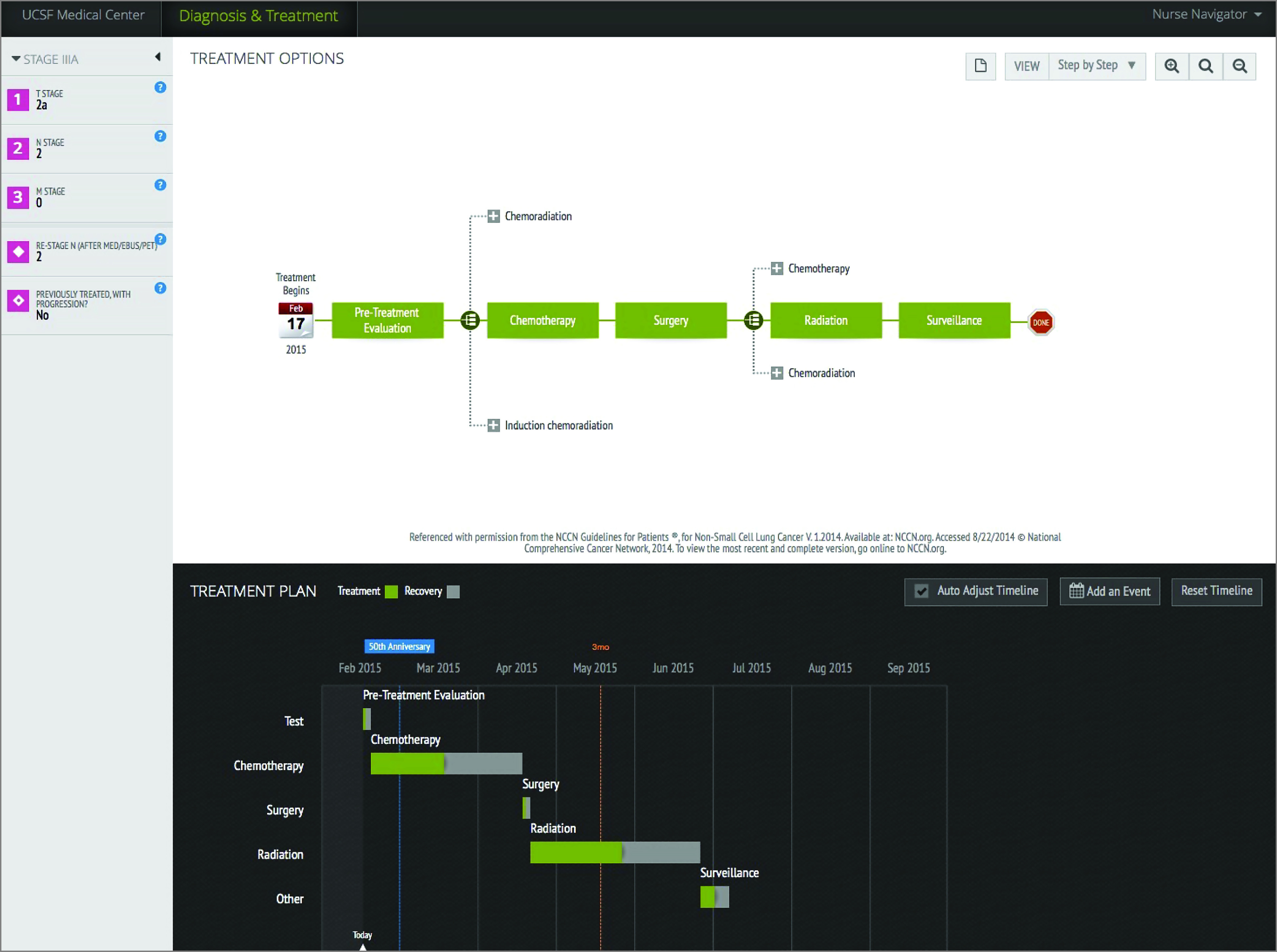
Example of the Web-Based Tool Interface for a Patient With a T2aN2 Tumor Resected With Negative Margins The patient (or research coordinator) inputs clinical variables in the left panel. Based on that information, treatment options consistent with National Comprehensive Cancer Center (NCCN) guidelines and the timeline representation of the overall treatment course are generated. UCSF indicates University of California, San Francisco.

An interactive web-based interface allowed patients, with assistance from a trained research coordinator, to enter their individual clinical, radiographic, and pathologic characteristics and then explore, in a structured manner, treatment combinations and sequences based on the NCCN guidelines (Figure 1). The decision tree created an interactive calendar-like timeline that allowed patients to map out their sequence of treatments over time.

We established 6 benchmarks of quality care derived from the guidelines that were evaluated in a binary manner: (1) documented smoking cessation counseling in active smokers, (2) adjuvant chemotherapy for patients with stage IB to IIB NSCLC after surgery, (3) pathologic mediastinal staging in patients with stage III NSCLC before surgery, (4) pathologic mediastinal staging in patients with stage III NSCLC before nonsurgical management, (5) definitive chemoradiotherapy for patients with stage III NSCLC not undergoing surgery, and (6) molecular testing for epidermal growth factor receptor and anaplastic lymphoma kinase alterations for patients with stage IV NSCLC. We performed a retrospective review of 157 consecutive patients meeting enrollment criteria and seen by the group in the 8 months before the trial to determine baseline levels of guideline concordance. Two independent reviewers (S.Y.W. and S.S.Y.) compared the rates of guideline concordance in the retrospective cohort vs the trial patients.

Six patient-reported instruments were used to evaluate decisional conflict, decision-making preferences, quality of life, and satisfaction with decisions. The Decisional Conflict Scale (DCS)^12^ consists of 5 subscales evaluating whether a patient feels informed, supported, uncertain, effective in decision-making, and clear regarding their values on a scale of 0 to 100, with higher values representing the most positive levels. The Decision Making Preference Questionnaire^13,14^ asks patients to identify their preferred level of decision-making responsibility, ranging from “the doctor should make the decisions” to “I should make the decisions.” The Functional Assessment of Cancer Therapy–Lung (FACT-L) assesses lung cancer–specific quality of life.^15^ The Satisfaction With Decision is a 6-item questionnaire measuring satisfaction on a scale of 1 (lowest) to 5 (highest).^16^ Patients also completed a postconsultation questionnaire querying proposed treatments, satisfaction, understanding, and expectation of tolerating treatment (eAppendix in Supplement 2).

Following consent and before consultation, patients completed a baseline Decision Making Preference Questionnaire, DCS, and FACT-L and were guided through the web-based tool by a trained research coordinator (T.L.D.), who helped enter clinical information to create the decision tree. The research coordinator then attended the visit, with the tool displayed on a laptop for reference during the consultation. Following consultation, patients completed the DCS, Satisfaction With Decision, and postconsultation questionnaires. The preconsultation questionnaires took 20 to 30 minutes to complete, training with the tool took approximately 15 minutes, and the postconsultation questionnaires required 10 to 20 minutes. All interventions were completed on the day of consultation.

### Statistical Analysis

The Fisher exact test and the Cochrane-Armitage trend test for ordered factors were used to compare clinical variables and guideline benchmarks between the 2 cohorts. The Wilcoxon signed rank test was used to compare DCS scores before and after consultation with the web-based tool. There were 9 participants with missing survey results preventing calculation of the change in DCS score (9/76 [12%]) and 3 with missing FACT-L subscores (3/76 [4%]). Multiple imputation by chained equations using 20 imputation data sets was performed as a sensitivity analysis^17,18^ (eTable 1 in Supplement 2). Multivariable regression linear (for continuous outcomes) and logistic (for dichotomous outcomes) analysis was performed to evaluate the association between outcomes and clinical variables. Use of the web-based tool was log transformed (log 10 + 1) since the fit of the model was better based on the residual plot, and we exponentiated the results. For ease of interpretation, we also dichotomized patient satisfaction with decision as satisfied or neutral/unsatisfied. We evaluated each variable in a separate model as a potential confounder, and we did not identify confounders requiring adjustment based on a *P* value <.05. We forced potential confounders into the multivariable model: age or age as quadratic, race/ethnicity, and cancer stage. The magnitude of the association after adjusting for clinical variables is presented as an adjusted estimate for age, race/ethnicity, and stage with the associated 95% CI.

Analysis of guideline concordance was matched based on clinical criteria appropriate for the benchmark (ie, stage IB-IIB disease for guideline 2). Because of the limited numbers of patients, further matching or inverse probability weighting via propensity score to reduce potential differences between groups was not possible owing to low event rates (<10 events), particularly for benchmarks 1, 2, and 3. Analyses were performed using SPSS, version 25 (IBM Corp) and SAS, version 9.4 (SAS Institute Inc). Two-sided *P* values <.05 were considered statistically significant except for benchmark comparisons of quality care where a 2-sided *P* value <.017 was used based on the Bonferroni correction for multiple testing.

The sample size calculation is described in the protocol (Supplement 1). We expected that the clinical volume of 30 patients per month for 12 months would yield a potential enrollment pool of 360 patients. Anticipating a 30% decline, dropout, or ineligibility rate at the time of potential enrollment, at least 250 patients would be evaluable. Data analysis was conducted from July 19, 2019, to April 22, 2020.

## Results

Between February 23, 2015, and September 28, 2017, 76 patients with NSCLC were prospectively enrolled. The median age was 68 years (interquartile range [IQR], 41-87 years) and 44 were men (57.9%); most of the 76 patients had adenocarcinoma (59 [77.6%]) or squamous cell carcinoma (14 [18.4%]) and stage I (28 [36.8%]) or stage IV (23 [30.3%]) disease. In the comparator cohort of 157 patients, the median age was 66 years (IQR, 61-65 years), 91 patients (58.0%) were men, most had adenocarcinoma (105 [66.9%]) or squamous cell carcinoma (28 [17.8%]) and stage I (35 [22.3%]) or stage IV (64 [40.8%]) disease. Other characteristics of the 2 cohorts are presented in Table 1, and their treatments are reported in eTable 3 in Supplement 2. Enrollment into the trial was slow, with 5 to 6 patients accrued per month during peak enrollment in 2015. We closed the trial in September 2017.

**Table 1.** Patient and Treatment Characteristics of Trial vs Retrospective Cohorts

Variable	No. (%)^a^		*P* value
	Trial cohort (n = 76 unless otherwise noted)	Retrospective cohort (n = 157 unless otherwise noted)	
Age at diagnosis, median (range), y	68 (41-87)	66 (61-65)	.44
Age at study, median (range), y	68 (41-88)	NA	
Female	32 (42.1)	66 (42.0)	.99
Male	44 (57.9)	91 (58.0)	
Race/ethnicity			
White	51 (67.1)	83 (52.8)	.03
Asian	11 (14.5)	24 (15.3)	
Black	10 (13.2)	11 (7.0)	
Other/declined	4 (5.3)	38 (24.2)	
Hispanic	6 (7.9)	7 (4.5)	.46
History of tobacco use			
No	19 (25.0)	42 (26.8)	.78
Yes	57 (75.0)	115 (73.2)	
Active smoker	5 (6.6)	24 (15.3)	.11
Histologic characteristics			
Adenocarcinoma	59 (77.6)	105 (66.9)	.10
Squamous cell carcinoma	14 (18.4)	28 (17.8)	
Adenosquamous	2 (2.6)	7 (4.5)	
Not biopsied	1 (1.3)	1 (0.6)	
Other (large cell or NOS)	0	16 (1.2)	
T classification (AJCC, 7th ed)			
1 a/b	28 (36.8)	40 (25.5)	.16
2 a/b	20 (26.3)	50 (31.8)	
3	8 (10.5)	31 (19.7)	
4	20 (26.3)	30 (19.1)	
Unknown/missing	4 (5.3)	5 (3.2)	
N classification			
0	35 (46.1)	63 (4.1)	.25
1	6 (7.9)	15 (9.6)	
2	25 (32.9)	51 (32.5)	
3	8 (10.5)	27 (17.2)	
Nonregional node	2 (2.6)	0	
M classification			
M1a	11 (14.5)	15 (9.6)	.07
M1b	12 (15.8)	46 (29.3)	
AJCC 7th ed stage group			
IA	20 (26.3)	20 (12.7)	.007^b^
IB	8 (10.5)	15 (9.6)	
IIA	5 (6.6)	2 (1.3)	
IIB	4 (5.3)	8 (5.1)	
IIIA	8 (10.5)	33 (21.0)	
IIIB	8 (10.5)	15 (9.6)	
IV	23 (30.3)	64 (40.8)	
Quality of care benchmark, No./No. (%)			
1. Documented smoking cessation counseling or intervention in active smokers	4/5 (80.0)	1/24 (4.2)	<.001
2. Adjuvant chemotherapy for patients with stage IB-IIB disease following surgery^c^	0/7	7/11 (63.6)	.01
Negative margins	0/7	4/6 (66.7)	.02
Tumors >4 cm	0/4	3/5 (60.0)	.20
3. Pathologic mediastinal staging completed before surgery for patients with stage III disease	1/2 (50.0)	5/14 (35.7)	>.99
4. Pathologic mediastinal staging before initiation of treatment in patients with stage III disease not undergoing surgery	4/14 (28.6)	12/34 (35.3)	.75
5. Initial chemoradiotherapy for patients with stage III disease not undergoing surgery	9/14 (64.3)	22/34 (64.7)	>.99
6. Testing for EGFR or ALK alteration status in patients with stage IV disease^d^	21/23 (91.3)	49/60 (81.7)	.50
Nonsquamous cell histologic findings only	20/20 (100.0)	48/57 (84.2)	.10
Before systemic therapy	19/20 (95.0)	47/56 (83.9)	.28

Abbreviations: AJCC, American Joint Committee on Cancer; ALK, anaplastic lymphoma kinase; EGFR, epidermal growth factor receptor; NA, not applicable; NOS, not otherwise specified.

^a^ Percentages may not add to 100 due to rounding.

^b^ Quality of care benchmarks 2 to 6 were matched by relevant stage. There was no significant difference in stage among active smokers included in benchmark 1 (eTable 4 in Supplement 2).

^c^ Denominator is less than the total number of patients with stage IB to IIB disease because not all patients received surgery.

^d^ Four patients with stage IV disease in the retrospective cohort had limited records that prevented us from assessing EGFR/ALK alteration testing and systemic therapy use and therefore were excluded from this analysis.

Rates of guideline-concordant treatment in the cohorts are reported in Table 1; these cohorts are shown by ethnicity in eTable 5 in Supplement 2. Among self-declared active smokers in the trial, 80.0% (4 of 5) had documented smoking cessation counseling compared with 4.2% (1 of 24) of patients in the retrospective cohort (*P* < .001). The trial cohort also received less adjuvant chemotherapy after surgery among patients with stage IB to IIB disease (0 of 7 vs 63.6% [7 of 11]; *P* = .012). In post hoc subanalysis, this difference seemed primarily driven by patients who underwent surgery with negative margins (0 of 7) in the trial cohort compared with 4 of 6 patients (66.7%) in the comparator cohort (*P* = .02). While there was no overall difference in the rates of molecular testing for epidermal growth factor receptor and anaplastic lymphoma kinase alterations in those with stage IV disease (21 of 23 [91.3%] vs 49 of 60 [81.7%]; *P* = .50), and among those with non–squamous cell histologic characteristics, the change in rates of testing was not significant (20 of 20 [100%] vs 48 of 57 [84.2%]; *P* = .10). There was no significant change in the trial vs retrospective cohort in the rate of pathologic mediastinal staging in patients with stage III NSCLC undergoing surgery (50.0% [1 of 2] vs 35.7% [5 of 14]; *P* > .99) or nonoperative management (28.6% [4 of 14] vs 35.3% [12 of 34]; *P* = .75) and no significant difference in the rate of upfront chemoradiotherapy for those with stage III NSCLC not undergoing surgery (64.3% [9 of 14] vs 64.7% [22 of 34]; *P* > .99).

Half (51.3%) of the trial patients expressed on the Decision Making Preference Questionnaire that they and their physician should take equal part in the decision-making process (Table 2). The median FACT-L total score was 67 (IQR, 61-75). Based on the FACT-L, trial patients generally felt supported by their families and friends (median subscore, 20; IQR, 9) and believed that their diagnosis had a moderate association with their functional well-being (median subscore, 14; IQR, 8-17) (Table 2).

**Table 2.** Survey Results Before Consultation in Patients With Non–Small Cell Lung Cancer

Variable	Finding
Decision Making Preference Questionnaire, No./No. (%)^a^	
The doctor should make the decisions using all that’s known about the treatments	5/76 (6.6)
The doctor should make the decisions but strongly consider my opinion	12/76 (15.8)
The doctor and I should make the decisions together on an equal basis	39/76 (51.3)
I should make the decisions but strongly consider the doctor’s opinion	13/76 (17.1)
I should make the decision using all I know or learn about the treatments	2/76 (2.6)
Missing/selected multiple answers	5/76 (6.6)
Achieved agreement on treatment recommendation between patient and physician	42/45 (93.3)
Achieved agreement between treatment and observation	62/65 (95.4)
Functional Assessment of Cancer Therapy–Lung (n = 74), median (IQR)^b^	
Subscore	
Physical (possible range, 0-28)	8 (3-11)
Social (possible range, 0-28)	20 (15-24)
Emotional (possible range, 0-24)	8 (6-12)
Functional (possible range, 0-28)	14 (8-17)
Additional symptoms (possible range, 0-36)	16 (14-19)
Total score (possible range, 0-144)	67 (61-75)
Additional questions	
Smoking regret (possible range, 0-4)	4 (3-4)
Fatigue (possible range, 0-4)	2 (1-3)
Bone pain (possible range, 0-4)	0 (0-2)
Satisfaction with health care decision (possible range, 1-5) (n = 70)^c^	
I am satisfied that I am adequately informed about the issues important to my decision	4 (4-5)
The decision I made was the best decision possible for me personally	4 (3-5)
I am satisfied that my decision was consistent with my personal values	4 (3-5)
I expect to successfully carry out (or continue to carry out) the decision I made	4 (4-5)
I am satisfied that this was my decision to make	4 (4-5)
I am satisfied with my decision	4 (4-5)

Abbreviation: IQR, interquartile range.

^a^ Balanced set of 5 choices of decision-making style; patients select the answer that fits their preference the most.

^b^ Scored from 0 to 144, with best quality of life indicated by higher score.

^c^ Scored from 1 to 5, with 5 indicating highest satisfaction.

**Figure 2.**
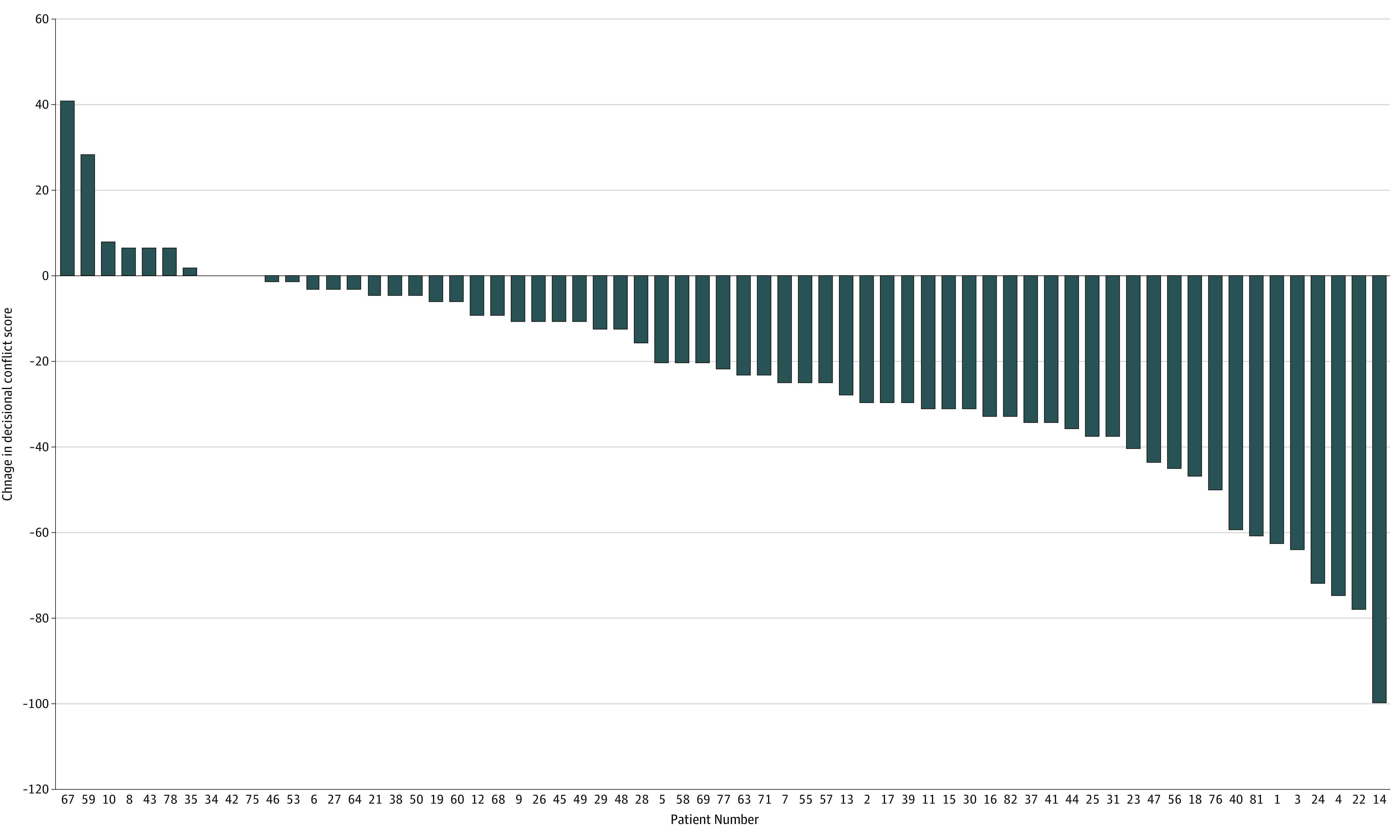
Waterfall Plot Showing Changes in Decisional Conflict Scale (DCS) Scores A negative change suggests decreased decisional conflict. Seven patients experienced an increase in their DCS score after consultation with the web-based tool, while 3 patients experienced no change in DCS score.

Following a guidelines-enhanced consultation, patients’ total DCS scores improved by a median decrease of 20 points (IQR, 3-34; *P* < .001) (Table 3). Significant decreases were seen across all subscales, with the largest decreases in the domains of feeling informed, clarity about personal values, and uncertainty (each with a median improvement of 25 points). Although most patients had a decrease in their DCS score, 7 patients’ (9.2%) scores increased and 3 scores (4.0%) were unchanged (Figure 2). The sensitivity analysis showed similar results (eTable 2 in Supplement 2).

**Table 3.** Changes in Decisional Conflict Scale Scores After Consultation Incorporating Web-Based Access to Non–Small Cell Lung Cancer Guidelines

Subscore	Median (IQR)^a^		
	Consultation score		Decrease in score
	Preconsultation	Postconsultation	
Informed	50 (27-58)	25 (0-33)	25 (0-42)
Clarity	50 (33-58)	25 (8-33)	25 (0-42)
Support	25 (17-50)	17 (0-25)	8 (0-25)
Uncertainty	50 (25-75)	25 (0-42)	25 (0-50)
Effective^b^	50 (25-50)	25 (0-31)	13 (0-44)
Total score	45 (29-57)	25 (8-33)	20 (3-34)

Abbreviation: IQR, interquartile range.

^a^ All findings significant at *P* < .001.

^b^ Skewness in the data affected the median value.

Patients satisfied with their decision (53 of 68 [77.9%]) had significant improvement in their DCS score after consultation compared with those who were not satisfied (adjusted odds ratio, 0.80; 95% CI, 0.71-0.91; *P* < .001). Sixty-four patients (84.2%) accessed the web-based tool a median of 3 times following their initial consultation (range, 0-20). On multivariable analysis, for a 10-unit improvement in DCS score, we expected a 6% increase in use of the tool following consultation while controlling for age, race/ethnicity, and stage (95% CI, 2%-9%; *P* = .002).

## Discussion

The findings of this study suggest feasibility and efficacy of a web-based, customizable decision support tool facilitating direct access to the NCCN guidelines. Exposure to the guidelines during consultation was associated with improvements in 2 indices of guideline-concordant care. Exposure was also associated with decreased decisional conflict and increased satisfaction with decision-making. In turn, decreased decisional conflict was associated with increased patient satisfaction with decisions. Patients with greater reductions in decisional conflict accessed the guidelines at a higher frequency after consultation.

Receipt of guideline-concordant care for lung cancer has been shown to improve median overall survival,^19,20^ but registry data suggest that only 20% to 50% of patients with NSCLC receive recommended therapy.^20,21^ There are many complex factors that may mediate delivery of guideline-concordant care,^22^ such as sex,^23^ race,^24^ ethnicity,^21^ insurance coverage,^25^ socioeconomic status,^25^ performance status,^26^ and patient preferences. Given our limited sample size, we could not explore these variables further.

Patients with stage IB to IIB cancer in our 2 cohorts were treated with the same standard of care, but the role of adjuvant chemotherapy following surgery for early-stage NSCLC remains controversial. According to 2 meta-analyses, the 5-year overall survival benefits may be on the order of 4% to 5%,^27,28^ but both of these studies included patients with stage I to stage III disease. However, mature results of the Cancer and Leukemia Group B (CALGB) 9633 trial demonstrated no survival benefit for patients with T2N0 NSCLC,^29^ and the JBR.10 trial, which stratified patients by nodal status at enrollment, found that adjuvant treatment with cisplatin plus vinorelbine did not improve overall survival in patients with stage IB cancer.^30^ Therefore, the NCCN guidelines for adjuvant therapy (consistent from v2.2013^31^ through v2.2018^32^) have recommended that, for stage IB-IIA (T2N0) cancers resected with negative margins, adjuvant chemotherapy should be considered for high-risk features, such as poorly differentiated tumors, vascular invasion, wedge resection, size greater than 4 cm, visceral pleural involvement, and unknown lymph node status. For patients with T2N0 cancers resected with a positive margin, further resection with or without chemotherapy or radiotherapy with or without chemotherapy (but with chemotherapy for patients with IIA disease) is recommended. In patients with N1 disease, adjuvant chemotherapy is recommended alone for negative margins or in conjunction with further resection or radiotherapy for microscopic or macroscopic residual tumor. In our study, in keeping with similar findings in low-risk breast cancer, patients’ exposure to a decision-support tool that recommends adjuvant treatment only for specific high-risk situations (in which the benefit of additional therapy is estimated to be low) was associated with a decision-making process that led to decreased use of the adjuvant therapy.^33^

The impact of patient-directed support tools has primarily been studied in patients with prostate or breast cancer^5,33^ or metastatic^34^ and locally advanced^35^ NSCLC; these tools have been shown to improve knowledge and decrease decisional conflict,^7^ and in patients with prostate and endometrial cancer considering adjuvant radiotherapy, higher DCS scores are associated with a likelihood of postponing decisions.^2^ Although a meta-analysis suggests that decision aids may not change a patient’s ultimate decision,^36^ use of the tools may improve the timeliness of decision-making, which impacts oncologic outcomes. In this trial, we found that the greatest decreases in decisional conflict were in the domains of feeling informed, clarity about personal values, and uncertainty, suggesting that patients gained knowledge about treatment options within the context of personal preferences and felt less uncertain about their decisions. A smaller reduction was seen in the domain of feeling supported owing to lower baseline scores before consultation (Table 3) and consistent with the FACT-L findings that most trial patients already felt supported by friends and family.

Although most randomized trials suggest that decision tools are not associated with increased anxiety for most patients,^9,36,37,38^ 7 patients (9.2%) in our trial reported increased decisional conflict after consultation. This finding is consistent with the work of Brundage et al,^39^ who found that 1 of 12 patients (8.3%) with stage III NSCLC experienced an increase in DCS scores after decision support. Increased decisional conflict may be an appropriately vigilant response to the presentation of information that challenges patients’ preconceptions.^40^ It is beyond this study’s scope to analyze patient or disease factors that might estimate increased conflict.

### Limitations

The primary limitation of this study is its small sample size due to unexpectedly slow accrual. This limitation was multifactorial but primarily owing to repeated changes in coordinators, resulting in 2 prolonged enrollment pauses that accounted for 17 of the 31 months that the trial was open. Another factor limiting enrollment was reliance on attending oncologists to screen eligible patients before consultation; the physicians insisted on performing this initial screening, which reduced eligibility. As such, the study was underpowered for the primary end point, although significant differences were still found.

This study was conducted at a tertiary academic medical center and may not be representative of the population at large. Decreased use of adjuvant chemotherapy may have been due to advanced-level discussions of toxic effects, options, and recurrence risk, but we cannot analyze this outcome further. Another potential confounder was the emergence of gene expression profiling of these patients with early-stage disease. However, molecular risk stratification was performed in only 3 patients in the trial cohort (2 by Encore Clinical, Brisbane, California, and 1 by VariStrat, Boulder, Colorado) and 7 patients in the retrospective cohort (6 by Pervenio Lung, Life Technologies Clinical Services Lat, West Sacramento, California, and 1 by Encore Clinical). Given these low numbers and the different assays used, we were unable to draw further conclusions regarding their impact.

The baseline rate of smoking cessation counseling in the retrospective cohort was low (4%). It is possible that some of the improvement seen during the trial was due to improved documentation by physicians (Hawthorne effect) or unquantified institutional factors, such as changes to the intake process. In addition, only 5 of 76 patients (6.6%) in the trial cohort identified as current smokers compared with 24 of 157 patients (15.3%) in the retrospective cohort, and it is possible that the rate of smoking was overestimated at baseline. It is difficult to identify how or why this overestimate may have occurred. In contrast, major management, such as pathologic mediastinal staging, and chemoradiotherapy for stage III patients did not change, reflecting a greater fixity and lack of efficacy of this tool in these areas.

One practical consideration is the resource-intensive nature of decision support. Data suggest that when patients self-administer decision support tools, there is limited improvement in knowledge^41^ or decisional conflict,^42,43^ suggesting that assistance resources are necessary. Also of concern is the effect of these tools on consultation time, although one Cochrane review suggested that the median consultation time was just 2.6 minutes longer with use of a decision aid.^36^ Then there is the burden of development, implementation, and maintenance of the tool to consider. Adjustments to this process would be needed to increase guideline access on a larger scale. During and after implementation of this study, some of our clinicians began drawing out diagrams or writing out the NCCN guidelines on paper during consultations. It may be that the online interactivity, further personalization, and real-time calendaring provided by our tool was less necessary than the basic guideline content. Nonguideline-based support could include providing a written summary or audio recording of patients’ consultations.^43,44^

As this trial was not randomized, it is not clear whether reductions in decisional conflict would have been seen after consultation alone. However, randomized trials have addressed this question and demonstrated decreased decisional conflict with the use of decision aids.^7^ Ours was a practical implementation study aimed at quality improvement rather than reestablishing the ethicality and validity of decision support. The magnitude and similar directionality of multiple reinforcing metrics testifies to the power of this intervention. While the tool in this study did not meet criteria for a decision aid,^44^ meta-analysis suggests that decision support has the ability to improve patient knowledge and decisional conflict at a level similar to that of decision aids.^37^ By using a standard national guideline in a supportive rather than directive manner, our intervention was acceptable to our clinicians.

Further research is needed to explore how best to implement decision support in routine clinical practice. Allocating limited clinic resources requires commitment, and a direct translation to cost savings or quantifiable improvement in quality is difficult to establish. Nonetheless, our study reinforces that there is a potential positive association with the patient’s experience and delivery of high-quality care.

## Conclusions

To our knowledge, this is the first study in patients with NSCLC that has suggested an association with guideline-concordant care using a patient-empowering educational approach. Patient exposure to the NCCN guidelines appears to be associated with increases in smoking cessation counseling and decreased use of adjuvant chemotherapy in patients with resected early-stage disease. Patients exposed to the tool appeared to have decreased decisional conflict and greater satisfaction with their decision. Despite the limitations of this study, these findings suggest that evidence-based decision and communication tools have potential to improve the quality of cancer care delivered.

## Notes

Supplement 1. **Trial Protocol**

Supplement 2. **eTable 1.** FACT-L Scores With and Without Multiple Imputation by Chained Equations Using 20 Imputation Datasets **eTable 2.** Decisional Conflict Scale (DCS) Scores Before and After Consultation Incorporating Web-Based Access to Non–Small Cell Lung Cancer Guidelines: A Pooled Analysis Using 20 Imputation Data Sets **eTable 3.** Treatment Including Surgery, Radiation and Chemotherapy in the Trial Cohort and Retrospective Cohort **eTable 4.** Distribution of Active Smokers by Clinical Stage **eTable 5.** Patients Included in Each Benchmark Analysis by Self-Reported Race/Ethnicity **eAppendix.** Post-Consultation Questionnaire
