# Supplementary material for: Evaluation of a National Comprehensive Cancer Network Guidelines–Based Decision Support Tool in Patients With Non–Small Cell Lung Cancer: A Nonrandomized Clinical Trial
Source: JAMA Netw Open. 2020 Sep 30;3(9):e209750. doi: 10.1001/jamanetworkopen.2020.9750 (PMC7527870; doi:10.1001/jamanetworkopen.2020.9750)
Supplement: Supplement 1. — Trial Protocol [file jamanetwopen-e209750-s001.pdf]

UCSF Helen Diller Family Comprehensive Cancer Center

Acceptability and Effectiveness of a Novel Internet-Based Decision-Support Aid Based on the NCCN Non-Small Cell Lung Cancer Patient Guidelines

Protocol Number: CC #14655
Version Number: 1.6

Version Date: 03-15-2016

IND Number: N/A

Principal Investigator (Sponsor-Investigator)
Dr. Sue S. Yom, M.D., Ph.D.
University of California, San Francisco
1600 Divisadero Street, San Francisco, CA 94143-1708
Telephone: 415-353-9893
Fax: 415-353-9883
E-mail: yoms@radonc@ucsf.edu

Co-Principal Investigator
Matthew Gubens, M.D.

Co-Investigators
Thierry Jahan, M.D.
David Jablons, M.D.
Alexander Gottschalk, M.D.
Collin Blakely, M.D.
Pierre Theodore, M.D.
Errol Bush, M.D.
Victoria Wang M.D.

Statistician
Vivian Weinberg, Ph.D.

Clinical Research Coordinator
Taylor Dunbar, B.A. University of California San Francisco
1600 Divisadero Street, Room H1031
San Francisco, CA 94143
Telephone: 415-353-9249

Revision History

Version 1.0	02-25-2014	Version 1.5	09-16-2015
Version 1.1	05-04-2014	Version 1.6	03-15-2016
Version 1.2	02-26-2015		
Version 1.3	04-30-2015		
Version 1.4	07-10-2015		

Proprietary and Confidential

The information in this document is considered privileged and confidential, and may not be disclosed to others except to the extent necessary to obtain Institutional Review Board approval and informed consent, or as required by Federal and State laws. Persons to whom this information is disclosed should be informed that this information is

privileged and confidential and that it should not be further disclosed.

Protocol Signature Page

Protocol No.: 1.6 **Version Date:** 03-15-2016

1. I agree to follow this protocol version as approved by the UCSF Protocol Review Committee (PRC), and Committee on Human Research (CHR.
2. I will conduct the study in accordance with applicable CHR requirements, Federal regulations, and state and local laws to maintain the protection of the rights and welfare of study participants.
3. I certify that I, and the study staff, have received the requisite training to conduct this research protocol.
4. I agree to maintain adequate and accurate records in accordance with CHR policies, Federal, state and local laws and regulations.

UCSF Principal Investigator / Study Chair

____
Printed Name

______________________________________ ________________
Signature Date

Abstract

Title	Acceptability and Effectiveness of a Novel Internet Based Decision Support Aid Based on the NCCN Non Small Cell Lung Cancer Patient Guidelines
Patient population	Newly diagnosed patients with NSCLC who have not yet started on any therapy
Rationale for Study	To improve the length and quality-of-life outcomes for people with non-small cell lung cancer (NSCLC) by enlisting and engaging patients in the dissemination of the NCCN guidelines. This is a two-year software development and implementation project to develop and field-test a decision aid based on the NCCN patient guidelines for NSCLC. The study goal is to improve the treatment decision-support process and experience for NSCLC patients in an academic medical center.
Primary Objective	The primary study outcomes will emerge from measurement of changes in overall practice patterns after implementation of the decision aid. Pre- and post-intervention data will be used to determine the level of difference before and after implementation of the decision aid into the thoracic oncology clinics. Six specific practice patterns will be queried in a binary (yes/no) manner:<br>1. All stages: smoking cessation discussion reinforced with a specific follow up plan and/or medication/patch/psychotherapy or counseling;<br>2. stage IB, IIA, IIB receiving surgery: adjuvant chemotherapy given after surgery;<br>3. stage III receiving surgery: pathologic staging of mediastinal nodal disease prior to initiation of therapies;<br>4. stage III not receiving surgery: pathologic staging of mediastinal nodal disease prior to initiation of therapies<br>5. stage III patients not receiving surgery: if concurrent chemotherapy and radiation are given upfront;<br>6. stage IV: molecular testing for EGFR and ALK mutations prior to initiation of systemic therapy.

Secondary Objectives	Secondary objectives are to:<br><br>1. Determine agreement between patient and physician post-consultation reports of treatments discussed and recommended, prognosis, and expected tolerance.<br>2. Determine correlations between overall and lung cancer-specific quality of life and decision-support preferences.<br>3. Determine correlations between overall and lung cancer-specific quality of life and decisional conflict.<br>4. Determine correlations between overall and lung cancer-specific quality of life and satisfaction with decision.<br>5. Determine correlations between decision-support preferences and decisional conflict and satisfaction with decision.<br>6. Determine correlations between decisional conflict and satisfaction with decision.
Study Design	This study involves the evaluation of the decision support tool Patients with Power, previously used with breast cancer patients, in the non-small cell lung cancer patient population.<br><br>1. Characterize physician and patient populations and treatment decisions in the UCSF thoracic oncology practices.<br><br>2. Establish the acceptability, feasibility, and effectiveness of a novel decision aid for decisions made about treatment for NSCLC.<br><br>3. Conduct an evaluation of changes in NCCN-guided practice patterns before and after implementation of the decision aid, with respect to 6 clinical scenarios:<br><br>• All patients: if smoking cessation intervention is provided at initial visit;<br>• Stage IB-IIB patients receiving surgery: if adjuvant chemotherapy is given after surgery;<br>• Stage III patients receiving surgery: if pathologic staging of mediastinal nodal disease is done prior to initiation of therapies;<br>• Stage III patients not receiving surgery: if pathologic staging of mediastinal nodal disease is done prior to initiation of therapies;<br>• Stage III patients not receiving surgery: if concurrent chemoradiation is given upfront;<br>• Stage IV patients: if molecular testing is done prior to initiation of systemic therapy.<br><br>4. Collect data to facilitate the design of future larger-scale evaluations, including assessments of physician-patient agreement and patients' levels of decisional conflict and satisfaction with decisions made as well as potential correlations to patient-reported quality to life.

Number of patients	250
Duration of Intervention	The patients will have access to the decision support tool indefinitely (lifetime) but collection of all study metrics will end at 12 months after the period of their first 2 initial consultations for NSCLC.
Duration of Follow up	Questionnaires will be completed at the time of initial consultation, with no planned follow up; PwP website-based metrics will be followed in aggregate for a maximum of 1 year.
Duration of study	Twenty four months:<br><br>Month 1-8: (1) Analyze existing practice patterns focused on 6 decision nodes derived from the NCCN decision tree for NSCLC. The study of practice patterns will be based on primarily on review of medical records by a trained research coordinator under Dr. Yom's direction. (2) Programming of NSCLC decision aid under Dr. Yom's direction, conducted with technical assistance as needed from PwP. The results will be reviewed by co-investigators for accuracy and ease of use.<br><br>Month 8-20: Conduct prospective trial of the decision aid, collecting data directly from patients over the course of their initial 2 consultations.<br><br>Month 20-24: Completion of data collection and data analysis activities.
Study Intervention	The interactive PwP interface allows structured exploration of choices, sequencing, and timing of therapies. The aid thus enables the patient to discuss proposed treatments with the oncologist (rather than assuming passive acceptance). Furthermore, the decision aid allows evolving display of the treatment choices and timeline/calendar in a graphical, simplified format during the discussion (rather than assuming absorption and recall of details of a verbal communication). We will collect pilot data related to the collaborative, patient-oriented approach facilitated by this decision aid.
Study Assessments	A total of five patient-reported instruments will be used to score overall and lung cancer-specific quality of life, decision-support preferences, decisional conflict, and satisfaction with decision:<br><br>1. Decision Support Preference Questionnaire (DMPQ)<br>2. FACT-L<br>3. FACT/NCCN-Lung Symptom Index (NCCN-FACT FLSI-17)<br>4. Decisional Conflict Scale (DCS)<br>5. Post-Consultation Questionnaire<br>6. Satisfaction with Health Care Decision (SWD)

Unique Aspects of this Study	The decision aid in this study will be developed in collaboration with a technology startup, Patients with Power (PwP). PwP has developed an innovative web-based software that displays patient-friendly content adapted from the NCCN guidelines. The interactive web interface allows physicians or patients to input an individual patient's characteristics and then, drawing from the NCCN-guidelines-based decision trees, to design a patient-specific decision tree accompanied by a timeline tying anticipated procedures to specific dates. At present, PwP has developed a proof of concept only for breast cancer, but PwP has shared proprietary data with the Principal Investigator establishing the feasibility of customizing the interface for NSCLC-related content.

List of Abbreviations

AE	Adverse event
CHR	Committee on Human Research (UCSF IRB)
CRC	Clinical Research Coordinator
CTMS	Clinical Trial Management System
FDA	Food and Drug Administration
GCP	Good Clinical Practice
HDFCCC	Helen Diller Family Comprehensive Cancer Center
ICH	International Conference on Harmonization
IRB	Institutional Review Board
NCI	National Cancer Institute
PRC	Protocol Review Committee (UCSF)

Table of Contents

Protocol Signature Page 2
Abstract 3
List of Abbreviations 7
1 Introduction 10
1.1 Background on Intervention 10
1.2 Patients with Power – An Innovative Decision Aid to Visualize NCCCN Guidelines 10
1.2.1 Patients' Reactions to PwP Proof of Concept 11
1.3 Rationale for the Proposed Study 12
2 Objectives of the Study 13
2.1 Primary 13
2.2 Secondary 13
2.3 Endpoints 14
2.3.1 Primary Endpoints 14
2.3.2 Secondary Endpoints 14
3 Study Design 14
3.1 Number of Subjects 14
3.2 Eligibility Criteria 14
3.2.1 Inclusion Criteria 14
3.2.2 Exclusion Criteria 15
3.3 Duration of Intervention 15
3.4 Duration of Follow Up 15
3.5 Study Timeline 15
3.5.1 Study Completion 16
4 Study Procedures and Observations 16
4.1 Schedule of Procedures and Observations 16
4.1.1 Pre-consultation Assessments 16
4.1.2 Patients with Power Training 16
4.1.3 Post-consultation Assessments 16
5 Reporting and Documentation of Results 17
5.1 Evaluation of PwP 17
6 Statistical Considerations and Evaluation of Results 19
6.1 Study Endpoints 19
6.1.1 Study Design 19
6.2 Determination of Sample Size and Accrual Rate 20
6.2.1 Sample Size and Power Estimate 20
6.2.2 Accrual estimates 21
6.3 Analyses Plans 21
6.3.1 Primary Analysis (or Analysis of Primary Endpoints) 21
6.3.2 Secondary Analysis (or Analysis of Secondary Endpoints) 21

7 Study Management 21
7.1 Pre-study Documentation 21
7.2 Institutional Review Board Approval 22
7.3 Informed Consent 22
7.4 Changes in the Protocol 22
7.5 Data Storage and Confidentiality 22
7.6 Record Keeping and Record Retention 23
8 Protection of Human Subjects 23
8.1 Protection from Unnecessary Harm.. 23
8.2 Protection of Privacy.. 24
References 25
Appendix 1 Questionnaires 27

1 Introduction

1.1 Background on Intervention

Many physicians are reluctant to disclose specific details to cancer patients, such as risks of treatment, prognosis and treatment alternatives.[1-4] Communication is complicated by the stigma and fear associated with cancer,[5] the complexity of the information, and uncertainty about the course of the disease and benefits of treatment.[6,7] Discussions of treatment options are detailed and prolonged, leading to information loss and misunderstandings. However, the literature shows that providing detailed information to patients has not been associated with adverse psychological sequelae and, in some studies, it has contributed to decreased anxiety and increased treatment adherence.

Most educational interventions for cancer patients aim to help manage side effects, decrease anxiety, and increase compliance.[8-13] Recently, decision aids have been developed, to help patients go beyond traditional educational interventions by engaging them more directly in the decision process.[14] Major types of decision aids are interactive videos, personalized audiotapes, or booklets. In its systematic review, the Cochrane Collaboration found that these interventions improved knowledge and encouraged more appropriate use of surgical interventions.[15]

A few studies have explored decision aids used jointly and interactively by physicians and patients during a consultation. Whelan et al. found that a Decision Board intervention for breast cancer patients was associated with increased knowledge and use of breast-conserving surgery.[16-18] Siminoff et al. tested Adjuvant Online with early-stage breast cancer patients and found that the intervention group was more knowledgeable and less likely to choose adjuvant therapy featuring very marginal benefit.[19-21]

This study will test an innovative, interactive decision aid designed to facilitate patient and oncologist decision-support as a team. Improved patient-physician communication is likely to yield benefits in knowledge, decision certainty, and satisfaction.

1.2 Patients with Power – An Innovative Decision Aid to Visualize NCCCN Guidelines

The decision aid in this study will be developed in collaboration with a technology startup, Patients with Power (PwP). PwP has developed an innovative web-based software that displays content excerpted from the NCCN patient guidelines. The interactive web interface allows physicians or patients to input an individual patient's characteristics and then, drawing from the NCCN-guidelines-based decision trees, to design a patient-specific decision tree accompanied by a timeline tying anticipated procedures to specific dates. At present, PwP has developed a proof of concept only for breast cancer, but PwP has shared proprietary data with the Principal Investigator establishing the feasibility of customizing the interface for NSCLC-related content.

The interactive PwP interface allows structured exploration of choices, sequencing, and timing of therapies. The aid thus enables the patient to discuss proposed treatments with the oncologist (rather than assuming passive acceptance). Furthermore, the decision aid allows evolving display of the treatment choices and timeline/calendar in a graphical, simplified format during the discussion (rather than assuming absorption and recall of details of a verbal communication). We will collect pilot data related to the collaborative, patient-oriented approach facilitated by this decision aid.

1.2.1 Patients' Reactions to PwP Proof of Concept

The PwP prototype decision aid has met with very positive responses in the breast cancer community:

> From a newly diagnosed patient: "[I[t is amazing. It seems that my surgeon is following the right course. She has not talked about chemotherapy yet since the surgery comes first and then pathology…. My surgeon is also interested in learning more about this software."

> From a 2-year survivor: "[K]nowing the NCCN guidelines as compared to what doctors are recommending is really valuable."

> From a 6-year survivor: "I love the concept and the sleek, clean, bright design…. With your drop down lists, it was a snap to pick the correct answers. Your site has the potential to be a great source of information for hungry patients. I would have loved to have had something like it when I went through treatment."

> From a friend of a patient: "She and I went on[line] together. She is not tech savvy so I wasn't sure how she would react. I am a geek so it makes perfect sense to me. She really liked it and was able to navigate around with ease…. We especially liked the fact that information was available at decision points where choices would need to be made. That makes the journey a whole lot less confusing."

> From a cancer survivor and activist: "If this existed when I'd been diagnosed, it would've been the compass to help guide and map out my journey."

Startup technology companies often launch prototypes in high-profile diseases such as breast cancer. This project will extend the innovation to a more marginalized and underserved population in NSCLC. While extending the concept to NSCLC, our project will preserve the core features of the decision aid, including the graphical visualization of the guidelines, the ease of navigation in the user interface, the personalization enabled by the drop-down boxes, and the ability to create, modify, and review multiple scenarios (see Figure 1).

Figure 1. Screenshot of Patients with Power decision aid, designed currently for patients with breast cancer.

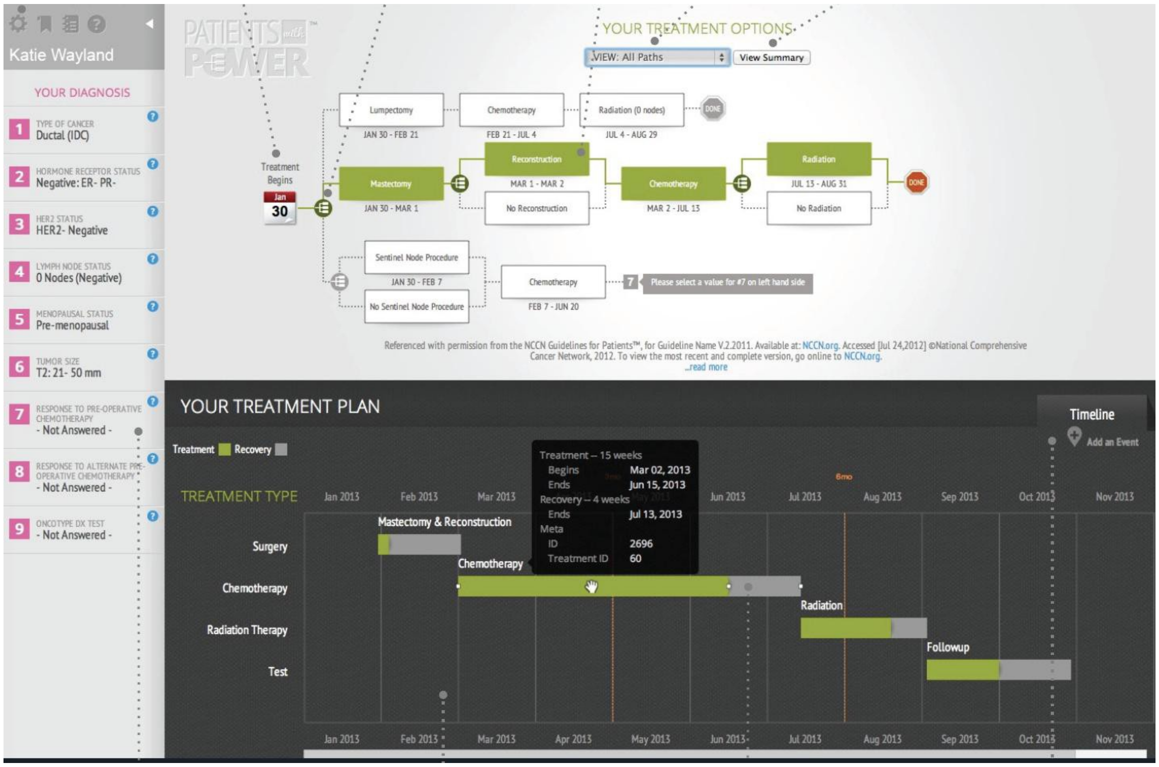

1.3 Rationale for the Proposed Study

This is a two-year career development project to develop and field-test a decision aid based on the NCCN patient guidelines for NSCLC. The study goal is to improve the treatment decision-support process in NSCLC patients in an academic medical center. If successful, this decision aid will be disseminated more broadly to community settings.

This study will consist of a development phase followed by an evaluation phase. After initial prototyping, refinement, coding, and implementation of the intervention, a study will be implemented using a pre/post design to estimate the effect size of the intervention with respect to disseminating NCCN-guided treatment choices (primary outcome) and to generate preliminary data about secondary outcomes.

Specifically, this project will:

1. Characterize physician and patient populations and treatment decisions in the UCSF thoracic oncology practices.

2. Establish the acceptability, feasibility, and effectiveness of a novel decision aid for decisions made about treatment for NSCLC.

3. Conduct an evaluation of changes in NCCN-guided practice patterns before and after implementation of the decision aid, with respect to 6 clinical scenarios:

1. All patients: if smoking cessation intervention is provided at initial visit;
2. Stage IB-IIB patients receiving surgery: if adjuvant chemotherapy is given after surgery;
3. Stage III patients receiving surgery: if pathologic staging of mediastinal nodal disease is done prior to initiation of therapies;
4. Stage III patients not receiving surgery: if pathologic staging of mediastinal nodal disease is done prior to initiation of therapies
5. Stage III patients not receiving surgery: if concurrent chemoradiation is given upfront;
6. Stage IV patients: if molecular testing is done prior to initiation of systemic therapy.

4. Collect data to facilitate the design of future larger-scale evaluations, including assessments of physician-patient agreement and patients' levels of decisional conflict and satisfaction with decisions made.

2 Objectives of the Study

2.1 Primary

The primary study outcomes will emerge from measurement of changes in overall practice patterns after implementation of the decision aid. Pre- and post-intervention data will be used to determine the level of difference before and after implementation of the decision aid into the thoracic oncology clinics. Six specific practice patterns will be queried in a binary (yes/no) manner:

1. All stages: smoking cessation discussion reinforced with a specific followup plan and/or medication/patch/psychotherapy or counseling;
2. Stage IB, IIA, IIB receiving surgery: adjuvant chemotherapy given after surgery;
3. Stage III receiving surgery: pathologic staging of mediastinal nodal disease prior to initiation of therapies;
4. Stage III not receiving surgery: pathologic staging of mediastinal nodal disease prior to initiation of therapies
5. Stage III patients not receiving surgery: if concurrent chemoradiation is given upfront
6. Stage IV: molecular testing for EGFR and ALK mutations prior to initiation of systemic therapy.

2.2 Secondary

Secondary objectives are to:

1. Determine agreement between patient and physician post-consultation reports of treatments discussed and recommended, prognosis, and expected tolerance.

2. Determine correlations between overall and lung cancer-specific quality of life and decision-support preferences.
3. Determine correlations between overall and lung cancer-specific quality of life and decisional conflict.
4. Determine correlations between overall and lung cancer-specific quality of life and satisfaction with decision.
5. Determine correlations between decision-support preferences and decisional conflict and satisfaction with decision.
6. Determine correlations between decisional conflict and satisfaction with decision.

2.3 Endpoints

2.3.1 Primary Endpoints

The practice pattern survey completed during the first 8 months will provide the baseline data that will be compared to results obtained after implementation of the decision aid.

2.3.2 Secondary Endpoints

A total of five patient-reported instruments will be used to score overall and lung cancer-specific quality of life, decision-support preferences, decisional conflict, and satisfaction with decision:

1. Decision Support Preference Questionnaire (DMPQ)
2. FACT-L
3. ACT/NCCN-Lung Symptom Index (NCCN-FACT FLSI-17)
4. Decisional Conflict Scale (DCS)
5. Post-Consultation Questionnaire
6. Satisfaction with Health Care Decision (SWD).

3 Study Design

3.1 Number of Subjects

The total number of participants to be enrolled is 250. The expected number of participants to be enrolled per quarter is 60-65.

3.2 Eligibility Criteria

The patient must be thoroughly informed about all aspects of the study, including the study visit schedule and required evaluations and all regulatory requirements for informed consent. The written informed consent must be obtained from the patient prior to enrollment. The following criteria apply to all patients enrolled onto the study unless otherwise specified.

3.2.1 Inclusion Criteria

1. Over age of 18

2. Able to provide informed consent
3. Able to use a web-based interface
4. Histologically proven or clinically apparent diagnosis of non-small cell lung cancer. Eligible NSCLC histological types include:
   - Adenocarcinoma
   - Squamous Cell Carcinoma
   - Large Cell Carcinoma
   - Adenosquamuos Carcinoma
5. Newly diagnosed, with new primary occurrence of NSCLC, or diagnosed with a new recurrence or new progression of existing disease, and not yet treated for the new problem.
6. Being seen in consultation at the UCSF thoracic oncology clinics

3.2.2 Exclusion Criteria

1. Unable to fill out questionnaires
2. Already treated for the current diagnosis of a new primary occurrence of NSCLC, or already treated for the new recurrence or new progression of existing disease.

3.3 Duration of Intervention

The patients will have access to the decision support tool indefinitely (lifetime) but collection of all study metrics will end at 12 months after the period of their first 2 initial consultations for NSCLC.

3.4 Duration of Follow Up

Questionnaires will be completed at the time of initial consultation, with no planned follow up; PwP website-based metrics will be followed in aggregate for a maximum of 1 year.

3.5 Study Timeline

Month 1-8:

(1) Analyze existing practice patterns focused on 6 decision points derived from the NCCN decision tree for NSCLC (see Main Scientific Question). The study of practice patterns will be based on direct observation of new patient visits coded by a trained research coordinator and review of medical records.

(2) Programming of NSCLC decision aid by a research fellow under my direction, conducted with technical assistance as needed from PwP. The results will be reviewed by co-investigators for accuracy and ease of use.

Month 8-20: Conduct prospective trial of the decision aid.

Month 21-27: Completion of data collection and data analysis activities.

First Participant in (estimated date of first participant enrolled): from 6/1/2014 to 2/23/2015

Last Participant in (estimated date of last participant enrolled): from 5/31/2015 to 8/1/2015

Last Participant Last Visit (estimated date of completion of Therapy for all participants enrolled): from 5/31/2015 to 8/1/2015

3.5.1 Study Completion

Completion of data collection and data analysis activities will be completed within 4 months after the study is closed to accrual.

4 Study Procedures and Observations

4.1 Schedule of Procedures and Observations

4.1.1 Pre-consultation Assessments

The pre-consultation questionnaires will take about 20-30 minutes to complete.

- Decision Support Preference Questionnaire (DMPQ)
- FACT-L
- NCCN-Functional Assessment of Cancer Therapy Lung Symptom Index-17 (NFLSI-17).
- Decisional Conflict Scale (DCS): The DCS will be completed before and after the patient's PwP training session.

4.1.2 Patients with Power Training

The patient will be trained on the use of the Patients with Power software by the Clinical Research Coordinator. The training will take approximately 15 minutes.

4.1.3 Post-consultation Assessments

The post-consultation questionnaires, completed by the patient and their oncologist, will take about 10-20 minutes to complete.

- Decisional Conflict Scale (DCS): The DCS will be also be completed before the patient's PwP training session.
- Post-Consultation Questionnaire
- Satisfaction with Health Care Decision (SWD).

Procedure	Clinical Visit
Informed consent	X
Decision Support Preference Questionnaire (DMPQ)	X[1]
FACT-L Questionnaire	X[1]
FACT/NCCN-Lung Symptom Index (NCCN-FACT FLSI-17) Questionnaire	X[1]
Decisional Conflict Scale (DCS) Questionnaire	X[1,2]
PwP Training Session	X
Post-Consultation Questionnaire	X[2]
Satisfaction with Health Care Decision (SWD) Questionnaire	X[2]

[1]pre consultation questionnaire
[2]post consultation questionnaire

5 Reporting and Documentation of Results

5.1 Evaluation of PwP

1. Decision Support Preference Questionnaire (DMPQ): The Decision Support Preference Questionnaire (DMPQ) is a brief assessment of patients' preferred style in decision making with their physician. Patients check off the single statement out of five that best describes their preferred level of shared decision making responsibility. The Decision Support Preference Questionnaire (DMPQ) will be completed *once before the consultation.*

2. FACT-L: The FACT-L is a 44-item self-report instrument that measures multidimensional quality of life, focused on common concerns of lung cancer patients measured on a 5-point Likert scale. It includes domains of Physical Well Being, Emotional Well Being, Social Well Being, and Functional Well Being. Available in eight languages, it is currently being used in several Phase II and III lung cancer clinical trials (Cella DF, Bonomi AE, Lloyd SR, Tulsky DS, Kaplan E, Bonomi P. Reliability and validity of the Functional Assessment of Cancer Therapy-Lung (FACT-L) quality of life instrument. Lung Cancer. 1995 Jun;12(3):199-220). The FACT-L will be completed *once before the consultation.*

3. NCCN-Functional Assessment of Cancer Therapy Lung Symptom Index-17 (NFLSI-17): The NCCN-FACT FLSI-17 is a 17-item patient-reported questionnaire that indexes the symptoms most highly valued by physicians, nurses, and patients with advanced-stage lung cancer.[25] It employs a five-point Likert scale and is scored from 0-68. It includes three subscales, Disease-Related Subscale (DRS), Treatment Side Effects (TSE), and Functional Well-Being (FWB). A unique aspect of this instrument is the

separation of disease-related and treatment-related effects. The NFLSI-17 will be completed *once before the consultation.*

4. Decisional Conflict Scale: The Decisional Conflict Scale (DCS) is a scale designed to measure patients' uncertainty in making health-related decisions, factors contributing to uncertainty, and patients' perceived effective decision-support. The DCS has 16 items and uses a five-point Likert scale. The scale is especially designed to help provide information about the efficacy of decision aids.[22] It has been used in a variety of cancer prevention and treatment-related settings. The DCS will be completed *before and after the consultation.*

5. Post-Consultation Questionnaire: The Post-Consultation Questionnaire will be completed by the patient and their oncologist *once after the consultation.* It includes the following components:

   a. Treatment Recommendations and Decisions: Treatments discussed, recommended, and chosen by the patient and their oncologist will be recorded. Options may include surgery, radiation therapy, chemotherapy, targeted therapy, observation, hospice, no treatment, or unresolved. The oncologist and patient will also be asked for an estimation of the tolerance of the treatment. This information will be obtained from the patient and oncologist *immediately after the consultation.*

   b. Prognosis Estimates: Estimates of prognosis with no treatment and with the recommended treatment will be obtained from the patient and oncologist *immediately after the consultation.*

   c. Program Evaluation: The patient will be asked to score the extent to which this process increased their understanding of their cancer treatment options and confidence in their oncologist *immediately after the consultation*.

6. Satisfaction with Health Care Decision (SWD): Although the DCS will provide a general measure of satisfaction with the medical encounter, it does not specifically query the patient's satisfaction with the health care decision itself. The Patient Satisfaction with Health Care Decision (SWD) is a six-item scale with excellent reliability and good discriminate validity. The instrument was validated using a sample of postmenopausal women specifically to evaluate the utility of patient decision aids.[14] The strength of the SWD is that it measures satisfaction with the decision made regardless of how good or bad the prognosis or health outcome might be for the individual patient. The SWD will be completed *once after the consultation.*

7. Acceptability: The PwP website will be monitored for usage *during the consultation and afterwards*. Patients will be given an account to enable continuous, unlimited access for the life of the grant. Usage statistics will be monitored only in aggregate terms, without reference to specific content accessed by individual patients. Repeat visiting to the website will be quantified to establish evidence of acceptance on the part of patients. Physician acceptance will be judged by rates of participation in using the decision aid during the consultation process.

6 Statistical Considerations and Evaluation of Results

6.1 Study Endpoints

Primary Endpoints: The practice pattern survey completed during the first 8 months will provide the baseline data that will be compared to results obtained after implementation of the decision aid.

Secondary Endpoints: A total of five patient-reported instruments will be used to score overall and lung cancer-specific quality of life, decision-support preferences, decisional conflict, and satisfaction with decision:

Decision Support Preference Questionnaire (DMPQ)

FACT-L

FACT/NCCN-Lung Symptom Index (NCCN-FACT FLSI-17)

Decisional Conflict Scale (DCS)

Post-Consultation Questionnaire

Satisfaction with Health Care Decision (SWD).

6.1.1 Study Design

This study involves the evaluation of the decision support tool Patients with Power, previously used with breast cancer patients, in the non-small cell lung cancer patient population.

1. Characterize physician and patient populations and treatment decisions in the UCSF thoracic oncology practices.

2. Establish the acceptability, feasibility, and effectiveness of a novel decision aid for decisions made about treatment for NSCLC.

3. Conduct an evaluation of changes in NCCN-guided practice patterns before and after implementation of the decision aid, with respect to 6 clinical scenarios:

All patients: if smoking cessation intervention is provided at initial visit;

Stage IB-IIB patients receiving surgery: if adjuvant chemotherapy is given after surgery;

Stage III patients receiving surgery: if pathologic staging of mediastinal nodal disease is done prior to initiation of therapies;

Stage III patients not receiving surgery: if pathologic staging of mediastinal nodal disease is done prior to initiation of therapies;

Stage III patients not receiving surgery: if concurrent chemoradiation is given upfront;

Stage IV patients: if molecular testing is done prior to initiation of systemic therapy.

4. Collect data to facilitate the design of future larger-scale evaluations, including assessments of physician-patient agreement and patients' levels of decisional conflict and satisfaction with decisions made as well as potential correlations to patient-reported quality to life.

6.2 Determination of Sample Size and Accrual Rate

6.2.1 Sample Size and Power Estimate

We expect that the clinical volume of 30 patients/month for 12 months will yield a potential enrollment pool of 360 patients. Anticipating a 30% decline, dropout, or ineligibility rate at the time of potential enrollment, then at least 250 patients should be evaluable. The sample size was determined based on the primary outcomes of changes in practice patterns. For each disease stage, a test of at least a 25% improvement using the decision aid compared with the pre-intervention results in the proportion of patients satisfying the stage-specific criterion listed above will be carried out (see Primary Endpoint for practice pattern metrics). In addition, the entire study sample will be evaluated to test for a change in smoking cessation plans. Patients will be included in up to 3 individual analyses. The maximum sample size for any one test is based upon assuming a null hypothesis of 50% in the pre-intervention cohort. This estimate for the null hypothesis will be modified using the results obtained prior to use of the decision aid (Months 1-8). Based upon the one-sample binary exact test with power set at 90% and a 2-sided type I error of 0.017 (adjusted for up to 3 tests for all patients), 53 patients would be required for each test. The test would reflect the actual accrual by stage with the use of the decision aid. With a fixed type I error of 0.017, this means for any individual test that the sample size may differ from 53, and the null hypothesis may differ from 50% and result in reduced power but will be at least 80%. For example, if adjuvant chemotherapy is given after surgery to 40% of stage IB and II patients determined from the pre-intervention assessment, then a test to detect a 50% improvement to 60% will require 70 patients with 80% power and 88 patients with 91% power. Similarly, if 60% of patients satisfy the smoking cessation criterion during the

pre-intervention interval, a 50% improvement will require 32 patients with 92% power, but a 15% improvement with 90% power requires 135 patients across all disease stages. It is therefore not anticipated that the comparisons will require more than the expected enrollment of 250 patients.

6.2.2 Accrual estimates

The total number of participants to be enrolled is 250. The expected number of participants to be enrolled per quarter is 60-65

6.3 Analyses Plans

6.3.1 Primary Analysis (or Analysis of Primary Endpoints)

The major focus of the trial analysis will be to determine if there is a statistically discernible effect for the decision aid in influencing practice patterns before and after implementation of the decision aid. The rates of NCCN adherence by stage will be an important covariate. The current sample size now reflects the ability to detect a moderate effect size for decision aid interactions with outcomes. More detailed analysis to correlate correlations between changes in practice patterns to other variables will be carried out with logistic regression, with special modifications for patient sociodemographic and clinical characteristics (e.g., nodes, tumor size, histology).

6.3.2 Secondary Analysis (or Analysis of Secondary Endpoints)

In addition to the primary analysis, additional analyses will be carried out to determine correlations between sociodemographic and clinical variables, similarity in physician and patient post-consultation reports, quality of life scores, decision-support preferences, decisional conflict, and satisfaction with decision.

All data analyses will be conducted by first using exploratory data techniques to examine univariate and bivariate relationships for statistical significance. Multivariate models will be constructed using statistical criteria, plausibility of associations, the existence of a priori hypotheses, and proposed causal pathways. Chi-square, non-parametric and log-linear techniques will be used for categorical or ordinal data, and t-tests, where appropriate, for continuous data. Multivariate models will use the observed likelihood ratio statistic as a statistical index of model fit. Observed effect sizes and their precision (expressed as 95% confidence intervals) will be more important components of these analyses than the associated p-values.

7 Study Management

7.1 Pre-study Documentation

This study will be conducted in accordance with the ethical principles that have their origin in the Declaration of Helsinki as stated in 21 CFR §312.120(c)(4); consistent with GCP and all applicable regulatory requirements.

Before initiating this trial, the Investigator will have written and dated approval from the Institutional Review Board for the protocol, written informed consent form, subject recruitment materials, and any other written information to be provided to subjects before any protocol related procedures are performed on any subjects.

The Investigator must comply with the applicable regulations in Title 21 of the Code of Federal Regulations (21 CFR §50, §54, and §312), GCP/ICH guidelines, and all applicable regulatory requirements. The IRB must comply with the regulations in 21 CFR §56 and applicable regulatory requirements.

7.2 Institutional Review Board Approval

The protocol, the proposed informed consent form, and all forms of participant information related to the study (e.g. advertisements used to recruit participants) will be reviewed and approved by the UCSF CHR (UCSF Institutional Review Board). Prior to obtaining CHR approval, the protocol must be approved by the Helen Diller Family Comprehensive Cancer Center Site Committee and by the Protocol Review Committee (PRC). The initial protocol and all protocol amendments must be approved by the IRB prior to implementation.

7.3 Informed Consent

All participants must be provided a consent form describing the study with sufficient information for each participant to make an informed decision regarding their participation. Participants must sign the CHR-approved informed consent form prior to participation in any study specific procedure. The participant must receive a copy of the signed and dated consent document. The original signed copy of the consent document must be retained in the medical record or research file.

7.4 Changes in the Protocol

Once the protocol has been approved by the UCSF CHR, any changes to the protocol must be documented in the form of an amendment. The amendment must be signed by the Investigator and approved by PRC and the CHR prior to implementation.

If it becomes necessary to alter the protocol to eliminate an immediate hazard to patients, an amendment may be implemented prior to CHR approval. In this circumstance, however, the Investigator must then notify the CHR in writing within five (5) working days after implementation. The Study Chair and the UCSF study team will be responsible for updating any participating sites.

7.5 Data Storage and Confidentiality

Patient confidentiality will be ensured at all times. Study results will not be released to participants. Patient identifiers will not be accessible to associated research scientists or clinicians conducting the research unless subsequently approved through the CHR. De-identified clinical and laboratory information may be shared with other investigators at other institutions. There are currently no plans to contact patients to inform them about

any finding of the study. Patient identifiers will not be made available to any other hospitals, insurers, or agencies. The results of studies emerging from this work may be published but individual patients will not be identifiable in these publications.

7.6 Record Keeping and Record Retention

The Principal Investigator is required to maintain adequate records.

The Principal Investigator is required to prepare and maintain adequate and accurate case histories that record all observations and other data pertinent to the investigation on each individual administered the intervention in the investigation. Case histories include the case report forms and supporting data (e.g., signed and dated consent forms and medical records, such as progress notes of the physician, the individual's hospital chart(s), and the nurses' notes. The case history for each individual shall document that informed consent was obtained prior to participation in the study.

Study documentation includes all CRFs, data correction forms or queries, source documents, Sponsor-Investigator correspondence, monitoring logs/letters, and regulatory documents (e.g., protocol and amendments, CHR correspondence and approval, signed patient consent forms).

Source documents include all recordings of observations or notations of clinical activities and all reports and records necessary for the evaluation and reconstruction of the clinical research study.

In accordance with FDA regulations, the investigator shall retain records for a period of 2 years following the date a marketing application is approved for the drug for the indication for which it is being investigated; or, if no application is to be filed or if the application is not approved for such indication, until 2 years after the investigation is discontinued and FDA is notified.

8 Protection of Human Subjects

8.1 Protection from Unnecessary Harm

Each clinical site is responsible for protecting all subjects involved in human experimentation. This is accomplished through the CHR mechanism and the process of informed consent. The CHR reviews all proposed studies involving human experimentation and ensures that the subject’s rights and welfare are protected and that the potential benefits and/or the importance of the knowledge to be gained outweigh the risks to the individual. The CHR also reviews the informed consent document associated with each study in order to ensure that the consent document accurately and clearly communicates the nature of the research to be done and its associated risks and benefits.

8.2 Protection of Privacy

Patients will be informed of the extent to which their confidential health information generated from this study may be used for research purposes. Following this discussion, they will be asked to sign the HIPAA form and informed consent documents. The original signed document will become part of the patient's medical records, and each patient will receive a copy of the signed document. The use and disclosure of protected health information will be limited to the individuals described in the informed consent document.

References

1. Silva MC, Sorrell JM. Enhancing comprehension of information for informed consent: a review of empirical research. *Irb.* Jan-Feb 1988;10(1):1-5.

2. Fitts WT, Jr., Ravdin IS. What Philadelphia physicians tell patients with cancer. *J Am Med Assoc.* Nov 7 1953;153(10):901-904.

3. Tuckett D, Williams A. Approaches to the measurement of explanation and information-giving in medical consultations: a review of empirical studies. *Soc Sci Med.* 1984;18(7):571-580.

4. Wu WC, Pearlman RA. Consent in medical decision making: the role of communication. *J Gen Intern Med.* Jan-Feb 1988;3(1):9-14.

5. What doctors tell patients with breast cancer about diagnosis and treatment: findings from a study in general hospitals. GIVIO (Interdisciplinary Group for Cancer Care Evaluation) Italy. *Br J Cancer.* Aug 1986;54(2):319-326.

6. Brewin TB. The cancer patient: communication and morale. *Br Med J.* Dec 24-31 1977;2(6103):1623-1627.

7. Frei E, 3rd. The National Cancer Chemotherapy Program. *Science.* Aug 13 1982;217(4560):600-606.

8. Dodd MJ. Cancer patients' knowledge of chemotherapy: assessment and informational interventions. *Oncol Nurs Forum.* Summer 1982;9(3):39-44.

9. Cassileth BR, Heiberger RM, March V, Sutton-Smith K. Effect of audiovisual cancer programs on patients and families. *J Med Educ.* Jan 1982;57(1):54-59.

10. Crosson K. Cancer patient education: what, where, and by whom? *Health Educ Q.* Spring 1984;10 Suppl:19-29.

11. Rimer B, Keintz MK, Glassman B. Cancer patient education: reality and potential. *Prev Med.* Nov 1985;14(6):801-818.

12. Beck S. Impact of a systematic oral care protocol on stomatitis after chemotherapy. *Cancer Nurs.* Jun 1979;2(3):185-199.

13. Jacobs C, Ross RD, Walker IM, Stockdale FE. Behavior of cancer patients: a randomized study of the effects of education and peer support groups. *Am J Clin Oncol.* Jun 1983;6(3):347-353.

14. Holmes-Rovner M. Evaluation standards for patient decision supports. *Med Decis Making.* Jan-Mar 1995;15(1):2-3.

15. Stacey D, Bennett CL, Barry MJ, et al. Decision aids for people facing health treatment or screening decisions. *Cochrane Database Syst Rev.* 2011(10):CD001431.

16. Whelan T, Levine M, Willan A, et al. Effect of a decision aid on knowledge and treatment decision making for breast cancer surgery: a randomized trial. *Jama.* Jul 28 2004;292(4):435-441.

17. Whelan T, Sawka C, Levine M, et al. Helping patients make informed choices: a randomized trial of a decision aid for adjuvant chemotherapy in lymph node-negative breast cancer. *J Natl Cancer Inst.* Apr 16 2003;95(8):581-587.

18. Whelan TJ, Loprinzi C. Physician/patient decision aids for adjuvant therapy. *J Clin Oncol.* Mar 10 2005;23(8):1627-1630.

19. Belkora JK, Hutton DW, Moore DH, Siminoff LA. Does use of the adjuvant! model influence use of adjuvant therapy through better risk communication? *J Natl Compr Canc Netw.* Jul 1 2011;9(7):707-712.

20. Peele PB, Siminoff LA, Xu Y, Ravdin PM. Decreased use of adjuvant breast cancer therapy in a randomized controlled trial of a decision aid with individualized risk information. *Med Decis Making.* May-Jun 2005;25(3):301-307.

21. Siminoff LA, Gordon NH, Silverman P, Budd T, Ravdin PM. A decision aid to assist in adjuvant therapy choices for breast cancer. *Psychooncology.* Nov 2006;15(11):1001-1013.

22. O'Connor AM. Validation of a decisional conflict scale. *Med Decis Making.* Jan-Mar 1995;15(1):25-30.

23. Holmes-Rovner M, Kroll J, Schmitt N, et al. Patient satisfaction with health care decisions: the satisfaction with decision scale. *Med Decis Making.* Jan-Mar 1996;16(1):58-64.

24. Cella DF, Tulsky DS, Gray G, et al. The Functional Assessment of Cancer Therapy scale: development and validation of the general measure. *J Clin Oncol.* Mar 1993;11(3):570-579.

25. Yount S, Beaumont J, Rosenbloom S, et al. A brief symptom index for advanced lung cancer. *Clin Lung Cancer.* Jan 2012;13(1):14-23.

26. Cassileth BR, Zupkis RV, Sutton-Smith K, March V. Information and participation preferences among cancer patients. *Ann Intern Med.* Jun 1980;92(6):832-836.

27. Strull WM, Lo B, Charles G. Do patients want to participate in medical decision making? *Jama.* Dec 7 1984;252(21):2990-2994.

Appendix 1a Questionnaires - Patient

Decision Making Preference Questionnaire

After they have all the information they need about their illness and possible treatments, some patients prefer to leave decisions about their treatment up to their doctor, while others prefer to participate in these decisions. Please check the statement that best describes what you believe would be ideal.

- ☐ The **doctor** should make the decisions using all that's known about the treatments.
- ☐ The **doctor** should make the decisions but strongly consider my opinion.
- ☐ The **doctor and I** should make the decisions together on an equal basis.
- ☐ **I** should make the decisions, but strongly consider the doctor's opinion.
- ☐ **I** should make the decisions using all I know or learn about the treatments.

FACT-L (Version 4)

Below is a list of statements that other people with your illness have said are important. **Please circle or mark one number per line to indicate your response as it applies to the <u>past 7 days</u>.**

	**<u>PHYSICAL WELL-BEING</u>**	**Not at all**	**A little bit**	**Some-what**	**Quite a bit**	**Very much**
GP1	I have a lack of energy	0	1	2	3	4
GP2	I have nausea	0	1	2	3	4
GP3	Because of my physical condition, I have trouble meeting the needs of my family	0	1	2	3	4
GP4	I have pain	0	1	2	3	4
GP5	I am bothered by side effects of treatment	0	1	2	3	4
GP6	I feel ill	0	1	2	3	4
GP7	I am forced to spend time in bed	0	1	2	3	4

	**<u>SOCIAL/FAMILY WELL-BEING</u>**	**Not at all**	**A little bit**	**Some-what**	**Quite a bit**	**Very much**
GS1	I feel close to my friends	0	1	2	3	4
GS2	I get emotional support from my family	0	1	2	3	4
GS3	I get support from my friends	0	1	2	3	4
GS4	My family has accepted my illness	0	1	2	3	4
GS5	I am satisfied with family communication about my illness	0	1	2	3	4
GS6	I feel close to my partner (or the person who is my main support)	0	1	2	3	4
Q1	*Regardless of your current level of sexual activity, please answer the following question. If you prefer not to answer it, please mark this box* ☐ *and go to the next section.*					
GS7	I am satisfied with my sex life	0	1	2	3	4

FACT-L (Version 4)

Please circle or mark one number per line to indicate your response as it applies to the <u>past 7 days</u>.

	**<u>EMOTIONAL WELL-BEING</u>**	**Not at all**	**A little bit**	**Some-what**	**Quite a bit**	**Very much**
GE1	I feel sad	0	1	2	3	4
GE2	I am satisfied with how I am coping with my illness	0	1	2	3	4
GE3	I am losing hope in the fight against my illness	0	1	2	3	4
GE4	I feel nervous	0	1	2	3	4
GE5	I worry about dying	0	1	2	3	4
GE6	I worry that my condition will get worse	0	1	2	3	4

	**<u>FUNCTIONAL WELL-BEING</u>**	**Not at all**	**A little bit**	**Some-what**	**Quite a bit**	**Very much**
GF1	I am able to work (include work at home)	0	1	2	3	4
GF2	My work (include work at home) is fulfilling	0	1	2	3	4
GF3	I am able to enjoy life	0	1	2	3	4
GF4	I have accepted my illness	0	1	2	3	4
GF5	I am sleeping well	0	1	2	3	4
GF6	I am enjoying the things I usually do for fun	0	1	2	3	4
GF7	I am content with the quality of my life right now	0	1	2	3	4

FACT-L (Version 4)

Please circle or mark one number per line to indicate your response as it applies to the <u>past 7 days</u>.

	**<u>ADDITIONAL CONCERNS</u>**	**Not at all**	**A little bit**	**Some-what**	**Quite a bit**	**Very much**
B1	I have been short of breath	0	1	2	3	4
C2	I am losing weight	0	1	2	3	4
L1	My thinking is clear	0	1	2	3	4
L2	I have been coughing	0	1	2	3	4
B5	I am bothered by hair loss	0	1	2	3	4
C6	I have a good appetite	0	1	2	3	4
L3	I feel tightness in my chest	0	1	2	3	4
L4	Breathing is easy for me	0	1	2	3	4
Q3	Have you ever smoked?<br>No ___ Yes ___ If yes:					
L5	I regret my smoking	0	1	2	3	4

Decisional Conflict Scale (DCS)

	Strongly Agree	Agree	Neither Agree Nor Disagree	Disagree	Strongly Disagree
I know which options are available to me.	☐	☐	☐	☐	☐
I know the benefits of each option.	☐	☐	☐	☐	☐
I know the risks and side effects of each option.	☐	☐	☐	☐	☐
I am clear about which benefits matter most to me.	☐	☐	☐	☐	☐
I am clear about which risks and side effects matter most to me.	☐	☐	☐	☐	☐
I am clear about which is more important to me (the benefits or the risks and side effects).	☐	☐	☐	☐	☐
I have enough support from others to make a choice.	☐	☐	☐	☐	☐
I am choosing without pressure from others.	☐	☐	☐	☐	☐
I have enough advice to make a choice.	☐	☐	☐	☐	☐
I am clear about the best choice for me.	☐	☐	☐	☐	☐
I feel sure about what to choose.	☐	☐	☐	☐	☐
This decision is easy for me to make.	☐	☐	☐	☐	☐
I feel I have made an informed choice.	☐	☐	☐	☐	☐
My decision shows what is important to me.	☐	☐	☐	☐	☐
I expect to stick with my decision.	☐	☐	☐	☐	☐
I am satisfied with my decision.	☐	☐	☐	☐	☐

AM O’Connor, Decisional Conflict Scale. ©1993 [updated 2005]. Available from www.ohri.ca/decionaid.
