# Supplementary material for: Evaluation of a National Comprehensive Cancer Network Guidelines–Based Decision Support Tool in Patients With Non–Small Cell Lung Cancer: A Nonrandomized Clinical Trial
Source: JAMA Netw Open. 2020 Sep 30;3(9):e209750. doi: 10.1001/jamanetworkopen.2020.9750 (PMC7527870; doi:10.1001/jamanetworkopen.2020.9750)
Supplement: Supplement 2. — eTable 1. FACT-L Scores With and Without Multiple Imputation by Chained Equations Using 20 Imputation Datasets eTable 2. Decisional Conflict Scale (DCS) Scores Before and After Consultation Incorporating Web-Based Access to Non–Small Cell Lung Cancer Guidelines: A Pooled Analysis Using 20 Imputation Data Sets eTable 3. Treatment Including Surgery, Radiation and Chemotherapy in the Trial Cohort and Retrospective Cohort eTable 4. Distribution of Active Smokers by Clinical Stage eTable 5. Patients Included in Each Benchmark Analysis by Self-Reported Race/Ethnicity eAppendix. Post-Consultation Questionnaire [file jamanetwopen-e209750-s002.pdf]

Supplementary Online Content

Wu SY, Lazar AA, Gubens MA, et al. Evaluation of a National Comprehensive Cancer Network guidelines–based decision support tool in patients with non–small cell lung cancer: a nonrandomized clinical trial. *JAMA Netw Open*. 2020;3(9):e209750.
doi:10.1001/jamanetworkopen.2020.9750

eTable 1. FACT-L Scores With and Without Multiple Imputation by Chained Equations Using 20 Imputation Datasets

eTable 2. Decisional Conflict Scale (DCS) Scores Before and After Consultation Incorporating Web-Based Access to Non–Small Cell Lung Cancer Guidelines: A Pooled Analysis Using 20 Imputation Data Sets

eTable 3. Treatment Including Surgery, Radiation and Chemotherapy in the Trial Cohort and Retrospective Cohort

eTable 4. Distribution of Active Smokers by Clinical Stage

eTable 5. Patients Included in Each Benchmark Analysis by Self-Reported Race/Ethnicity

eAppendix. Post-Consultation Questionnaire

This supplementary material has been provided by the authors to give readers additional information about their work.

© 2020 Wu SY et al. *JAMA Network Open.*

eTable 1. FACT-L scores with and without multiple imputation by chained equations using 20 imputation datasets

FACT-L Subscore	Original score, median (IQR)	Pooled scores, median (IQR)
**Physical subscore**	7 (3-11)	7 (2-11)
**Social subscore**	20 (15-24)	20 (15-24)
**Emotional subscore**	8 (6-11)	8 (6-10)
**Functional subscore**	15 (12-20)	15 (11-20)
**Additional subscore**	15 (14-19)	15 (14-19)
**Total subscore**	67 (61-75)	67 (61-75)

© 2020 Wu SY et al. *JAMA Network Open.*

eTable 2. Decisional Conflict Scale (DCS) scores before and after consultation incorporating web-based access to non-small cell lung cancer guidelines: a pooled analysis using 20 imputation data sets

**Subscore**	**Pre-consultation, median (IQR)**	**Post-consultation, median (IQR)**	**Change, median (IQR)**	**P value**
Informed subscore	50 (25-58)	25 (0-33)	25 (0-42)	< 0.001
Clarity subscore	50 (33-58)	25 (8-33)	22 (0-50)	< 0.001
Support subscore	25 (17-50)	17 (0-25)	8 (0-25)	< 0.001
Uncertainty subscore	50 (25-75)	25 (0-45)	25 (0-50)	< 0.001
Effective subscore	50 (25-50)	25 (0-40)	13 (0-44)	< 0.001
**Total score**	44 (30-53)	25 (9-33)	18 (5-34)	< 0.001

© 2020 Wu SY et al. *JAMA Network Open.*

eTable 3. Treatment including surgery, radiation and chemotherapy in the trial cohort and retrospective cohort

**Treatment**	**Trial cohort (N, %[a] of 76)**	**Pre-trial cohort (N, %[a] of 157)**
Surgical resection	14 (18.4%)	58 (36.9%)
Radiation to primary +/- nodes	40 (52.6%)	89 (56.7%)
Use of chemotherapy		
Neoadjuvant	7 (9.2%)	6 (3.8%)
Concurrent with radiation (+/- neoadjuvant or adjuvant)	13 (17.1%)	41 (26.1%)
Adjuvant	1 (1.3%)	15 (9.6%)
Neoadjuvant and Adjuvant	0 (0.0%)	3 (1.9%)
Palliative	13 (17.1%)	40 (25.5%)

[a] Percentages may not add to 100 due to rounding
*As this was not intended to be compared between the two cohorts, no p-values provided

eTable 4. Distribution of active smokers by clinical stage

Stage	Trial cohort (N, %[a] of 76)	Pre-trial cohort (N, %[a] of 157)	P-value
**I**	2 (2.6%)	5 (3.2%)	0.40
**II**	1 (1.3%)	1 (0.6%)	
**III**	1 (1.3%)	11 (7.0%)	
**IV**	1 (1.3%)	7 (4.5%)	

© 2020 Wu SY et al. *JAMA Network Open.*

eTable 5. Patients Included in Each Benchmark Analysis by Self-reported Race/Ethnicity

Benchmark	Trial cohort	Pre-trial cohort	P - value
Benchmark 1: Documented smoking cessation counseling or intervention in active smokers			0.11
White	1	13	
Black	3	3	
Asian	0	2	
Other/Declined	1	6	
Total	**5**	**24**	
Benchmark 2: Adjuvant chemotherapy for patients with stage IB-IIB disease following surgery			0.43
White			
Black	5	9	
Asian	0	0	
Other/Declined	2	1	
Total	0	1	
	**7**	**11**	
Benchmark 3: Pathologic mediastinal staging completed prior to surgery for patients with stage III disease			0.68
White	2	8	
Black	0	2	
Asian	0	2	
Other/Declined	0	2	
Total	**2**	**14**	
Benchmark 4: Pathologic mediastinal staging prior to initiation of treatment in patients with stage III disease not undergoing surgery			0.38
White	10	12	
Black	3	3	
Asian	0	5	
Other/Declined	1	14	
Total	**14**	**34**	
Benchmark 5: Upfront chemoradiation for patients with stage III disease not undergoing surgery			0.55
White	10	12	
Black	3	3	
Asian	0	5	
Other/Declined	1	14	
Total	**14**	**34**	
Benchmark 6: Testing for EGFR or ALK alteration status in patients with stage IV disease			0.22
White			
Black	12	35	
Asian	2	3	
Other/Declined	7	11	
Total	2	11	
	**23**	**60**	

© 2020 Wu SY et al. *JAMA Network Open.*

eAppendix. Post-Consultation Questionnaire

1. Treatment options discussed:

□	Surgery	□	Observation
□	Radiation Therapy	□	Hospice
□	Chemotherapy	□	No Treatment
□	Don't Know	□	Targeted Therapy

2. Treatment recommended:

□	Surgery	□	Observation
□	Radiation Therapy	□	Hospice
□	Chemotherapy	□	No Treatment
□	Don't Know	□	Targeted Therapy

3. Treatment selected:

□	Surgery	□	Observation
□	Radiation Therapy	□	Hospice
□	Chemotherapy	□	No Treatment
□	Don't Know	□	Targeted Therapy

4. Survival prognosis with no treatment: ________ years ________months

5. Survival prognosis with recommended treatment: ________ years ________months

6. Expected tolerance of proposed treatment plan:

Not Tolerated	Poorly Tolerated	Don't Know	Somewhat Tolerated	Well Tolerated
o	o	o	o	o

7. Your satisfaction with the medical encounter:

Very Unsatisfied	Unsatisfied	Neutral	Satisfied	Very Satisfied
o	o	o	o	o

8. This process helped me understand my cancer treatment options better:

Strongly Disagree	Disagree	Neutral	Agree	Strongly Agree
o	o	o	o	o

© 2020 Wu SY et al. *JAMA Network Open.*

9. This process gave me more confidence in my doctor:

Strongly Disagree	Disagree	Neutral	Agree	Strongly Agree
o	o	o	o	o

© 2020 Wu SY et al. *JAMA Network Open.*
